# Water-Deficit Stress in the Epiphytic Elkhorn Fern: Insight into Photosynthetic Response

**DOI:** 10.3390/ijms241512064

**Published:** 2023-07-27

**Authors:** Jakub Oliwa, Andrzej Skoczowski, Grzegorz Rut, Andrzej Kornaś

**Affiliations:** Institute of Biology and Earth Sciences, Pedagogical University of Krakow, Podchorążych 2, 30-084 Kraków, Poland; andrzej.skoczowski@up.krakow.pl (A.S.); grzegorz.rut@up.krakow.pl (G.R.); andrzej.kornas@up.krakow.pl (A.K.)

**Keywords:** *Platycerium bifurcatum*, chlorophyll *a* fluorescence, fluorescence 77 K, gas exchange, leaf reflectance, photosynthesis

## Abstract

Progressive climate changes cause disturbance of water relations in tropical rainforests, where epiphytic ferns are an important element of biodiversity. In these plants, the efficiency of photosynthesis is closely related to the efficiency of water transport. In addition, due to the lack of contact with the soil, epiphytes are extremely susceptible to water-deficit stress. The aim of this experiment was to determine the response of the photosynthetic apparatus of *Platycerium bifurcatum* to a 6-week water deficit. The hydration and pigment composition of leaves were determined using reflectance spectroscopy and epifluorescence microscopy. Chlorophyll *a* fluorescence kinetics parameters, fluorescence induction curves (OJIP), low-temperature fluorescence curves at 77 K and proline concentration were analyzed at seven time points. After a decrease in leaf hydration by 10–15%, there were disturbances in the oxidation–reduction balance, especially in the initial photochemical reactions, a rapid decrease in plant vitality (PI) and significant fluctuations in chlorophyll *a* fluorescence parameters. The relative size of PSI antenna structures compared to PSII decreased in the following weeks of water deficit. Changes in photochemical reactions were accompanied by a decrease in gross photosynthesis and an increase in proline concentration. Changes in the functioning of photosynthesis light phase and the pigment composition of leaves are related to the resistance of elkhorn fern to long-term water deficit.

## 1. Introduction

Water deficiency is one of the key environmental factors limiting photosynthetic activity in vascular plants, especially ferns, whose leaves show less-resistant xylem to embolism compared to angiosperms [1,2,3]. Moreover, it inhibits photosynthesis by reducing the photosystem II (PSII) activity and causes an imbalance between the light-dependent reactions and dark reactions of photosynthesis. This leads to impaired transport and accumulation of assimilates [4,5]. When water deficit limits the assimilation of CO_2_, the excess of absorbed light energy can cause photodestruction of PSII components and reduce chlorophyll (Chl) content in the leaf tissue [6,7,8]. Water stress also increases the production of reactive oxygen species (ROS), which may contribute to temporary or permanent destruction of the photosynthetic apparatus, e.g., by hindering the synthesis of PSII reaction core [9,10].

Progressive climate change causes disturbance of water relations in the ecosystems of tropical rainforests. This results in the need to launch mechanisms preventing the negative effects of water deficit in plants [11]. The strategies used by ferns to protect photosynthesis are in this case various, due to different habitat preferences. This is especially noticeable between terrestrial and epiphytic forms [2,12]. Epiphytes are an ecological group that is particularly sensitive due to a lack of contact with the soil and naturally limited access to water [13,14]. For this reason, they have developed a number of morphological, anatomical and physiological adaptations. Even species that grow naturally in rainforests show some xerophytic features [1].

In epiphytes, the extended survival time under water-deficit stress is ensured by anatomical modification of the leaves in order to store water and increase resistance to desiccation, e.g., by increasing cuticle thickness [15]. On the other hand, the ability to tolerate low relative water content and long-term functioning with closed stomata seems to be crucial [12]. Ferns exhibit passive hydraulic control of stomata and differ from seed plants in this respect [16,17]. In addition, it has been shown that the photosynthetic capacity of epiphytic ferns is evolutionarily linked to traits associated with water transport capacity as well as specialized adaptations to its deficiency [12,15,18].

Epiphytes account for about 25% of the flora species of tropical forests [13]. In addition, nearly 1/3 of the fern species known to us belong to this ecological group [19]. This shows the significant role played by epiphytic ferns in rainforest ecosystems, whose species diversity is almost twice as high as that of bromeliads, which are a frequent subject of ecophysiological research [15]. Considering the key role of these species in maintaining the biodiversity of plants and invertebrates, as well as numerous threats caused by increasing anthropopressure, the current knowledge of the mechanisms of physiological response to abiotic stresses in ferns is still insufficient and significantly poorer compared to seed plants.

Research on the physiology of *Platycerium bifurcatum* under abiotic stress conditions has so far focused mainly on the photosynthetic response to high light, as well as the effects of ozone pollution [20,21,22]. The physiological response of this species to drought was determined only for the gametophyte stage [23]. In other species of epiphytic ferns growing under water stress, changes in gas exchange efficiency (especially stomatal conductivity and net photosynthesis) are relatively well known [2,12,24,25]; however, few ecophysiological studies take into account the impact of stress on the photosynthetic light phase [14,26]. However, the analysis of photochemical processes seems to be crucial for a full understanding of the photosynthetic apparatus response mechanism to water deficit, if we take into account that the efficiency of gas exchange is directly related to the production of assimilation forces in the light-dependent phase. Despite this, studies on ferns, in which PSII activity was determined, are usually limited only to the general assessment of the quantum efficiency of the photosystem, e.g., on the basis of the Fv/Fm index [14,26].

The aim of the study was to investigate the functioning of the photosynthetic apparatus in epiphytic fern *P. bifurcatum* during a 6-week water deficit and to determine the potential mechanisms of protection of the photosynthetic apparatus. It was determined to what extent water stress causes (i) disturbances in individual phases of electron transport in PSII and between PSII and PSI (ii), changes in the size of antenna structures in PSI and PSII, (iii) changes in the pigment composition of leaves and (iv) changes in photosynthesis rate (P*g*) and cellular respiration (R).

## 2. Results

### 2.1. Morphological and Anatomical Changes

**Figure 1:**
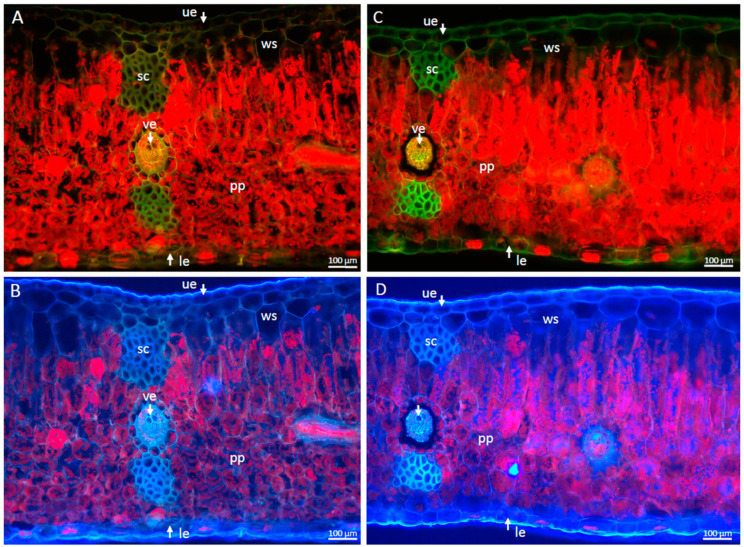
Cross-section of leaves of *Platycerium bifurcatum* observed using epifluorescence microscopy. (**A**,**B**): control plants, (**C**,**D**): plants after 6 weeks of dehydration. Red, green and blue colors correspond to autofluorescence of chlorophyll and cell walls, respectively; le—epidermis; pp—palisade parenchyma; sc—sclerenchyma; ue—upper epidermis; ws—water storage tissue; ve—vein.

Despite significant differences at the physiological level, no changes in leaf morphology and anatomy were noted between dehydration and control plants. There was no marked loss of turgor in the water-storing cells located directly below the upper epidermis. Under UV excitation (Figure 1B,D), a slight extinction of red autofluorescence is visible, associated with a decrease in Chl content in plants after 6 weeks of water deficit.

### 2.2. Changes in Hydration and Pigment Composition of Leaves

**Figure 2:**
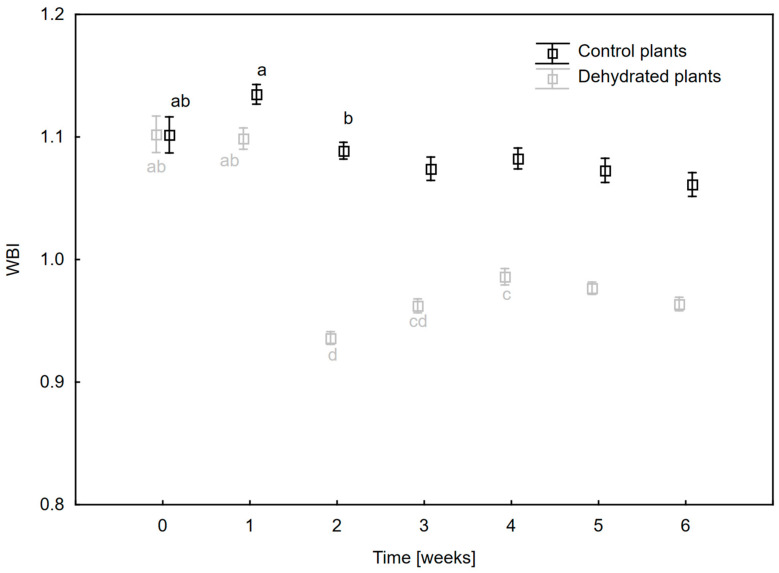
Changes in water band index (WBI) values in *Platycerium bifurcatum* leaves in the following 6 weeks after dehydration and in control plants. Mean values from 5 biological replicates ±SD, marked with different letters, differ significantly according to Tukey’s test, *p* ≤ 0.05.

Leaf hydration estimated on the basis of water band index (WBI) decreased during 6 weeks of plant growth under drought, but this decrease did not exceed 15% of the control value (Figure 2). The largest decrease in hydration occurred between the first and second week (W1 and W2) and then (despite slight fluctuations) remained at a similar level. Only slight fluctuations in the WBI values were observed in the control plants.

**Figure 3:**
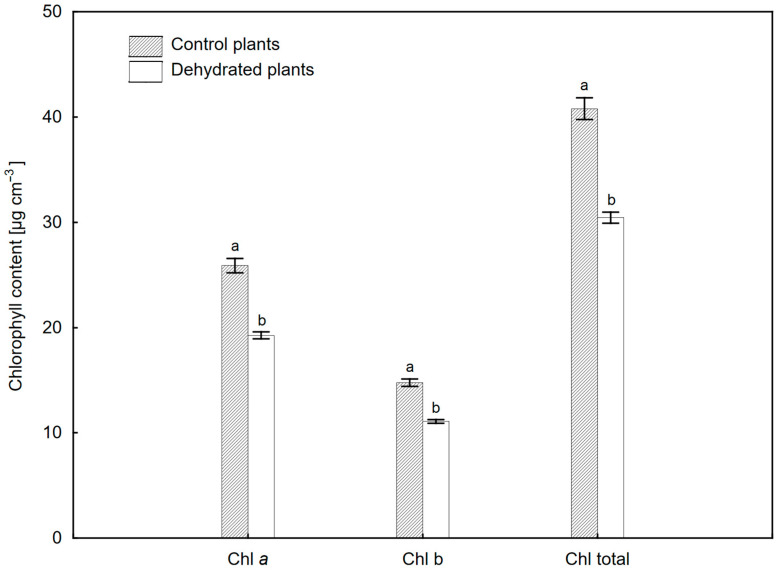
Chlorophyll (Chl) content in leaves of *Platycerium bifurcatum* in the 6 weeks after dehydration in control plants. Mean values from 5 biological replicates ±SD, marked with different letters, differ significantly according to Tukey’s test, *p* ≤ 0.05.

Chl content was significantly lower after 6 weeks of dehydration compared to control plants (Figure 3), but the Chl *a*/*b* ratio was similar (1.74—dehydrated plants 1.75—control plants). Changes in selected pigments in leaves during a 6-week dehydration period (W0–W6) are shown in Table 1. In the pool of anthocyanins (ARI1) and carotenoids (CRI1), a temporary decrease was observed between the first and second week of water deficit (W1 and W2), but the values of these parameters did not differ significantly between W0 and W6. Also, the structure insensitive pigment index (SIPI) value indicating the ratio of Chl to carotenoids (Car) did not change significantly during subsequent measurements (Table 1). One week after dehydration (W1), a significant reduction in the content of flavonols (FRI) as well as the photochemical reflectance index (PRI) was observed. The values of these parameters increased again in the second week; however, at the end of the experiment, they were significantly lower than the initial values. The data for the control plants (Table S1) indicate no significant differences in the pigment composition of leaves.

Leaf reflectance spectra 6 weeks after dehydration (Figure S1) revealed a decrease in the intensity of light reflection by leaves in the infrared range, associated with less tissue hydration. In the case of control plants, this decrease was not significant.

### 2.3. Chlorophyll a Fluorescence Analysis

**Figure 4:**
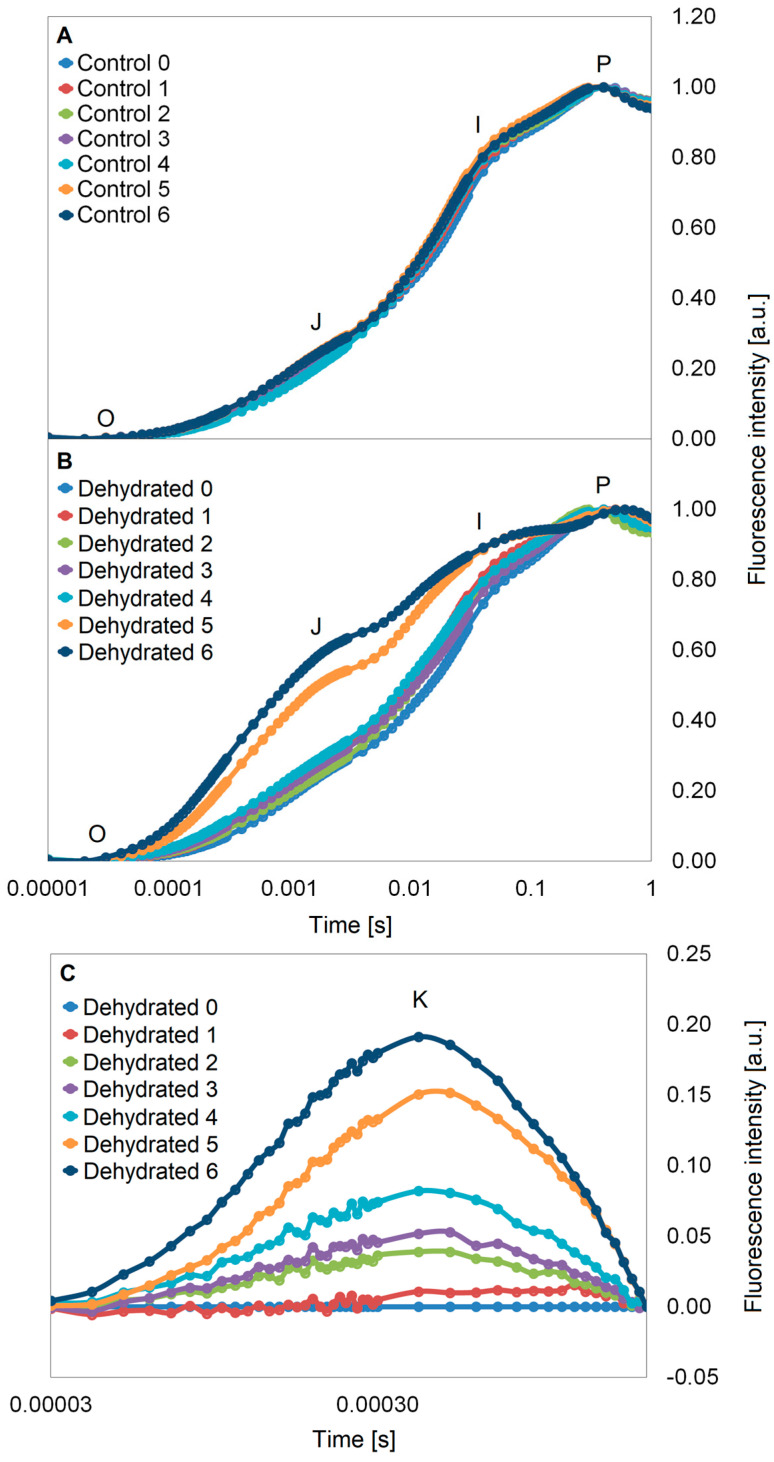
Double normalized chlorophyll *a* fluorescence induction curves (OJIP) in *Platycerium bifurcatum* leaves: (**A**)—in control plants and (**B**)—in the following 6 weeks after dehydration; (**C**)—differential curves of O–J phase with K bands in plants growing under water deficit. Mean values from 5 biological replicates.

The shape of the entire OJIP curves (from point O to point P) over four weeks was not different between dehydrated plants and control plants. Changes in the efficiency of electron transport within PSII took place after 5 weeks of dehydration (W5) which resulted in a significant increase in fluorescence (FL), especially in the O–J and J–I phases (Figure 4A,B). Disturbances in energy transport in PSII in W5 and W6 were observed as high FL values in the K band (Figure 4C). However, it should be noted that the K band was already visible in the second week of water deficit (W2) (Figure 4C). In addition, on the non-normalized curves for the last two dates, an increase in the Fm value was noticeable, and in W6, an increase in the F0 value was also observed, compared to W0 (Figure S2).

Changes in Chl *a* fluorescence parameters in *P. bifurcatum* before and during the 6-week water deficit are shown in Table 2 (control values Table S2). The dynamics of PSII functioning under drought stress were illustrated by the PI_total_ vitality index. The PI_total_ values decreased from the first week of water deficit (W1), and from the W5, they did not exceed 10% of the initial value (W0). In dehydrated plants, a statistically significant decrease in the values of Fv/Fm and Fv/F0 occurred in the second week of water deficiency (W2) and continued to decrease until W6. A downward trend was also observed in Am values. The size of the antenna for active reactive centers (RCs) increased (ABS/RC) in dehydrated plants in the following 6 weeks (Table 2). In the control plants, the values of the JIP test basic parameters did not change over time (Table S2).

In dehydrated plants, both energy trapping per single RC (TR_0_/RC) and total energy dissipation not trapped by the RCs (DI_0_/RC) increased from W2 and reached the highest values in W6 (Table 2). At the same time, the rate of electron transfer by the active RC (ET_0_/RC) after a slight, temporary increase returned to the initial values after 5 weeks of dehydration. In addition, the quantum yield of electron transport from Q_A_ to the PSI (RE_0_/RC) decreased permanently in W5. In the control plants, the values of specific energy fluxes per active RC remained at the same level (Di_0_/RC, RE_0_/RC) or fluctuated slightly (TR_0_/RC, ET_0_/RC) during the 6 weeks of the experiment. The values of all analyzed quantum yield parameters decreased gradually in the following 6 weeks after dehydration, which was not observed in the control plants. The quantum yield for the reduction of end electron acceptors at the PSI acceptor side (φRo) decreased in W1, while the quantum yield for electron transport from Q_A_- to plastoquinone (φEo) decreased significantly only in W5 (Table 2).

### 2.4. The Size of the PSI and PSII Antenna Complexes

**Figure 5:**
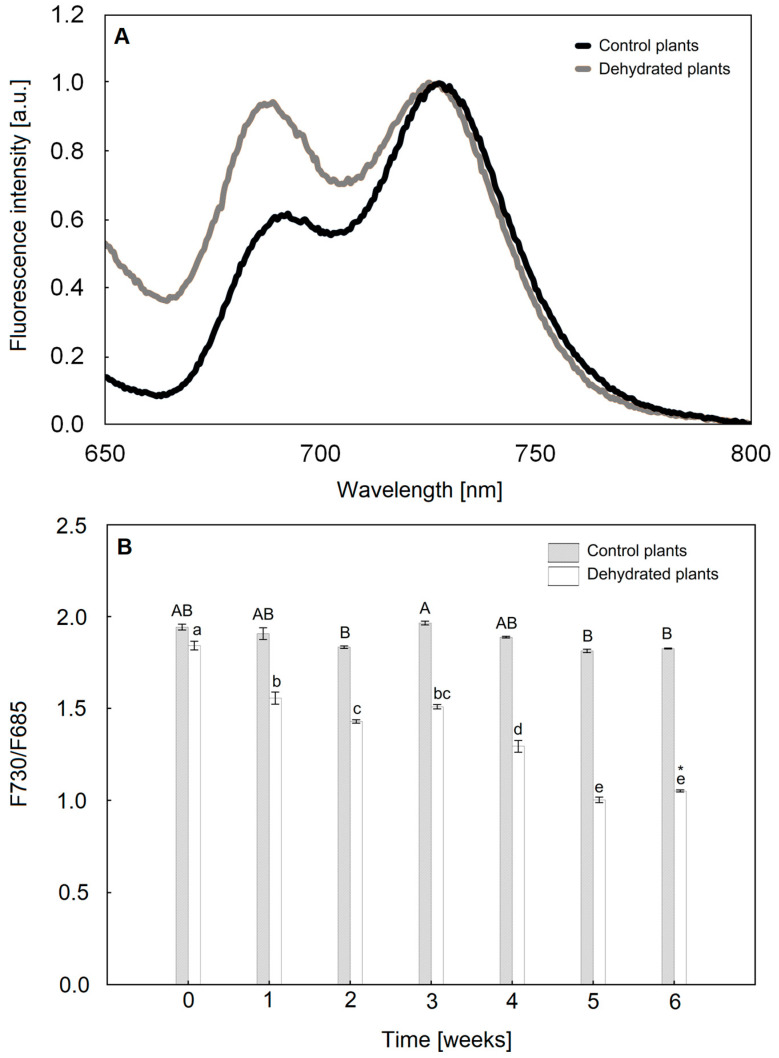
(**A**)—Low-temperature fluorescence curves at 77 K in *Platycerium bifurcatum* leaves in control plants and in the 6 weeks after dehydration; (**B**)—ratio of maximum PSI/PSII fluorescence values in control plants and in the following 6 weeks after dehydration. Mean values from 3 biological replicates ±SD marked with different letters differ significantly within treatment according to Tukey’s test, *p* ≤ 0.05.

Analysis of low-temperature fluorescence spectra at 77 K revealed a higher relative size of antenna structures concentrated around PSI (F730) compared to PSII components (F690) in control plants. However, with water deficiency after 6 weeks, comparable FL intensities were observed in bands from both photosystems (Figure 5A). In addition, in dehydrated plants, the maximum of both bands was shifted towards shorter wavelengths (blueshift), on average by 3.5 nm for PSII and 2.5 nm for PSI in relation to watered plants (Figure 5A). The relative size of PSI antenna structures in relation to PSII gradually decreases in the following weeks after dehydration (Figure S3A), as evidenced by a decrease in fluorescence emission in the band with a maximum at ca. 730 nm (Figure S3A). At the same time, in irrigated plants, the relative difference of Chl fluorescence emission for the main PSI and PSII bands did not change (Figure S3B). The ratio of maximum PSI to PSII fluorescence values (estimated on the basis of F730/F690 values) gradually decreased in dehydrated plants, assuming at the end of the experiment an average value 43% lower than the initial value and 42% lower than the control value measured at the same time point (Figure 5B).

### 2.5. Efficiency of Gas Exchange of Leaves

**Figure 6:**
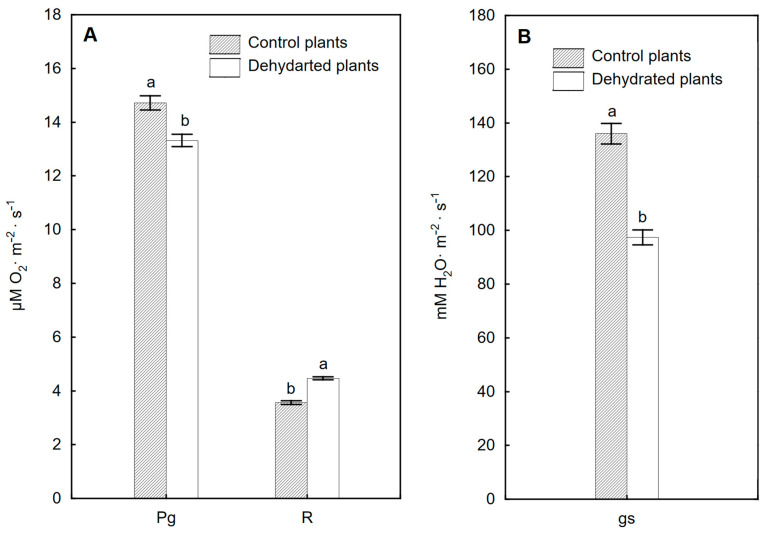
Gas exchange parameters in control plants and in the 6 weeks after dehydration: (**A**)—gross photosynthesis (Pg), (**B**)—cellular respiration (R), (**B**)—stomatal conductance (gs). Mean values from 3 biological replicates, with different letters within one parameter differing significantly according to Tukey’s test, *p* ≤ 0.05.

Gas exchange parameters measured after 6 weeks of drought for dehydrated and control plants are shown in Figure 6. In dehydrated plants, a decrease in gross photosynthesis rate was found (almost 10% on average) compared to the control. In contrast, respiration increased in dehydrated plants by an average of 25% compared to the control. The gs value decreased after 6 weeks of water deficit by 28% compared to the control.

### 2.6. Proline Content

**Figure 7:**
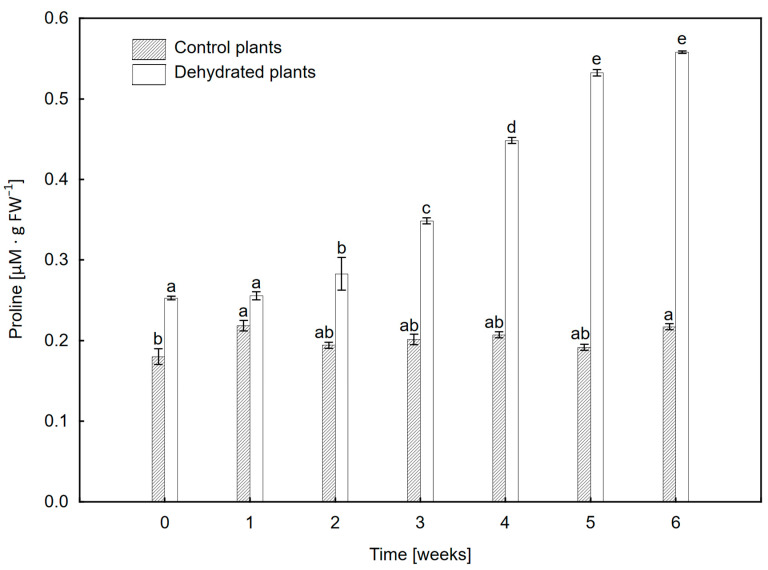
Proline changes in *Platycerium bifurcatum* leaves in control plants and in the following 6 weeks after dehydration. Mean values from 3 biological replicates ±SD, with different letters within treatments differing significantly according to Tukey’s test, *p* ≤ 0.05.

The proline content in the leaves of dehydrated plants increased systematically from week 2 and in week 6 it was more than twice as high as at the beginning of the experiment (Figure 7). At the same time, the proline content in control plants remained at a similar level.

## 3. Discussion

### 3.1. Morphological and Anatomical Features and Changes in the Content of Water and Pigments in Leaves

High leaf tolerance to water deficiency is common in epiphytic ferns [27]. Among ferns, the percentage of drought-tolerant species is much higher than in the case of seed plants [16]. In our study, we did not observe anatomical differences between dehydrated plants and watered plants. This is probably due to the xeromorphic characteristics of the leaves, which is an evolutionary adaptation to periodic water shortages. The leaves of mature *P. bifurcatum* sporophytes are up to 1.5 m long and approx. 2–3 mm thick, covered with a layer of cuticle and trichomes, which additionally protect the plant against water loss [28]. Such adaptations to periodic water shortages are also common in other epiphytic ferns, for which the presence of succulent leaves and the ability to tolerate low relative water content enable them to survive periods of a lack of water availability [12,14,17]. The morphological and anatomical structure of *P. bifurcatum* leaves explains the relatively small degree of water loss in tissues, which we observed during plant growth under drought based on the analysis of the WBI index (Figure 2) and reflectance spectra—Figure S1. High reflectance values in the NIR range are typical in plants in good physiological condition, which is conditioned by the spatial structure of leaf tissue. On the other hand, a decrease in the intensity of reflectance in the range of 950–970 nm indicates a water deficit [29].

In our study, the WBI values during the 6-week water-deficit period decreased by no more than 15% compared to the control (Figure 2). Numerous studies have shown that a decrease in leaf hydration results in low values of this parameter [30]. WBI shows a positive correlation with the RWC index, which is why it has been used many times to monitor changes in water content in plants [29,31,32]. Also, in other species of epiphytic ferns such as *Elaphoglosuum glaucum*, *E. petiolatum*, temporary drought stress did not result in a significant loss of leaf hydration or did not cause a decrease in RWC at all [14,33]. However, significant changes occurred in the pigment composition of *P. bifurcatum* leaves. After 6 weeks of drought, the Chl content decreased by about 25%. This effect has been observed in many species of tropical ferns, both during long and short periods of water shortage [14,25,26]. It is related to the progressive process of photooxidation and degradation of pigments [34,35,36].

The measurement of reflectance allows for a quick assessment of the pigment composition of leaves under environmental stress [37]. ARI1, CRI1 and FRI are sensitive indicators of changes in the content of anthocyanins, carotenoids and flavonols, increasing proportionally to the pigment concentrations in the tissue [38,39]. In our study, despite the initial slight decrease in ARI1 and CRI1 values, the content of anthocyanins and carotenoids returned back to the control level. This may suggest the involvement of anthocyanins and carotenoids in the adaptation of *P. bifurcatum* to the conditions of water deficit, which requires confirmation. Often, during abiotic stress, the decrease in the Chl content is accompanied by the production of pigments that perform photoprotective functions for PSII by absorbing excess energy, inhibiting the production of ROS and stabilizing chloroplast membranes [40,41]. In turn, the PRI value is correlated with zeaxanthin deepoxidation in the xanthophyll cycle and the efficiency of light use in the photosynthesis process [42].

### 3.2. Light-Dependent Reactions of Photosynthesis under Drought Stress

Chl *a* fluorescence kinetics analysis is often used to comprehensively assess the impact of environmental stress factors on light absorption and photosynthetic electron transport, as well as photochemical efficiency and communication between PSII units [43,44,45]. Thanks to the well-described methodology, the use of JIP test and analysis of Chl *a* fluorescence induction curves (OJIP) allows for the early detection of disturbances in the light phase of photosynthesis, which are the plants’ responses to abiotic stress, including water-deficit stress [46,47].

Analysis of OJIP curves revealed an increase in FL in the O–J phase, which proves a decrease in the overall absorptive capacity of the light-harvesting complex of PSII (LHCII) and a decrease in connectivity between PSII RCs [48,49]. The decrease in the efficiency of energy transfer between antenna complexes and PSII reaction centers is usually associated with changes in the structure of thylakoids during stress, as well as reorganization of PSII units [48,50]. In plants growing under water stress, rapid phosphorylation of LHCII proteins (such as Lhcb4) and simultaneous protein phosphatase-dependent dephosphorylation of PSII proteins (D1, D2) were observed, even in the absence of thylakoid damage [47]. The increase in FL intensity in the O–J phase and the appearance of K bands (Figure 4C) indicate more ungrouped PSII units in photosynthetic membranes and less activity of the oxygen-evolving complex in PSII [51]. The key changes in the shape of the OJIP curve occurred in the fifth week of plant growth under water-deficit conditions (Figure 4B). In turn, the increase in FL in the J–I phase suggests disturbances in the oxidation–reduction balance of the Q_A_ pool, similar to those observed in *P. bifurcatum* during high light stress [21]. In plants growing in natural conditions, the increase in FL in this phase may be the effect of multistress [52].

The decrease in PI_total_ (Table 2) suggests that the main disturbances in the light phase in *P. bifurcatum* plants growing under drought concern the efficiency of electron trapping as well as their transport beyond palstoquinone Q_A_ [47]. Reducing the efficiency of energy conversion in PSII leads to a decrease in plant vitality, significantly hindering the prevention of further symptoms of stress [53]. In our study, the PI_total_ decreased faster than the Fv/Fm parameter, which is most often used in ecophysiological studies of ferns [14,26,54]. Also, in two other epiphytic ferns (*Neottopteris nidus*, *Microsorum punctatum*), the Fv/Fm value was quite constant during 50 days of plant growth under water deficit [26]. On the other hand, in the ferns *E. luridum* and *Mohria caffrorum*, the PSII quantum yield and the electron transport rate through the photosystem decreased slightly only at below 70% RWC [14]. This confirms earlier reports that PI_total_ is a much more sensitive indicator of stress in epifitic fern *P. bifurcatum* than the assessment of PSII maximum photochemical efficiency [21]. The increase in the RC/ABS value suggests that there was an increase in the size of the PSII antenna per active RC [51]. This is related to the increasing degree of energy trapping per RC (TR_0_/RC—Table 2). Taking into account the reduction in the LHCII absorptive capacity, we hypothesize that the expansion of an active RC’s antenna and the increase in the efficiency of electron trapping is a form of adaptation of the photosynthetic apparatus to drought conditions, which needs to be confirmed in further studies.

Abiotic stress results in inhibition of electron transfer and energy imbalance between PSII and PSI. Our analysis of JIP test parameters indicated disturbances in energy transport within the PSII. In turn, the measurement of low-temperature fluorescence allowed for assessment of changes in the FL emission of both PSII and PSI antenna structures [55]. In control plants, the main FL bands were located at 690 nm for PSII and 730 nm. The relative size of PSI antenna structures in relation to PSII decreased during the 6 weeks in dehydrated plants, as evidenced by the values of F730/F690, and additionally, the maximum of both bands was shifted towards shorter wavelengths (blueshift) (Figure 5A,B). In turn, the loss of PSI core proteins determines the appearance of blueshift [56,57]. Thus, this confirms that water deficiency negatively affects LHCI antennas and PSI proteins.

### 3.3. Gas Exchange Regulation and Drought Adaptation Strategies

The most frequently observed effect of water deficiency in plants is the closing of stomata, reduction of stomatal conductivity and transpiration and, consequently, a decrease in photosynthetic activity. Measurements made on *P. bifurcatum* leaves after a 6-week drought revealed a decrease in gs by about one-third compared to the control and a reduction in Pg to about 10% of the control value. Also, among the six species of ferns (including two epiphytic) studied by McAdam and Brodribb [12], all of them closed their stomata at very low levels of water stress.

Water deficit caused a decrease in gross photosynthesis in *P. bifurcatum* plants, which was the result of stomatal closure as well as impaired functioning of PSI and PSII (Figure 6A). Prolonged water deficit may lead to a reduction in RubisCO activity or its content. This is manifested by a decrease in photosynthetic activity caused by a low electron transport rate in the photosynthetic light phase and limited carboxylation rate [2]. In addition, a decrease in net photosynthesis and transpiration caused by stomatal limitation was also noted in *P. bifurcatum* during other abiotic stresses, e.g., with increased ozone concentration, and was also associated with PSII damage [22].

Increase in proline content (Figure 7) confirms the activation of mechanisms related to counteracting stress in *P. bifurcatum* plants exposed to water deficit. Plant vegetation under stress induces the synthesis of compounds ensuring the stability of osmotic conditions, which allow for combating or mitigating the effects of stress [57,58,59]. The synthesis of proline and non-enzymatic antioxidants is to maintain the osmotic balance, ensure the structural integrity of the cell and enable the uptake of more water from the environment [60,61]. Therefore, it is one of the important elements of the strategy of protection against abiotic stress [61,62]. In particular, plants accumulate proline during a water deficit and drought [63,64], although this process is not always activated during short-term stress [60]. This shows the diversity of the mechanisms of osmoprotectant synthesis in response to abiotic stress, which, especially in ferns, are not well understood and require further research, taking into account various plant species inhabiting endangered ecosystems.

## 4. Materials and Methods

### 4.1. Plant Material

In the experiment, 10 mature, 8-year-old sporophytes of the fern *P. bifurcatum* from the own collection of the Pedagogical University of Krakow were used. The plants were grown under semi-controlled conditions in a climatic chamber located in a greenhouse under natural light, at a constant temperature of 23 °C day/16 °C night (photoperiod 16 h day/8 h night) and humidity (RH = 60% ± 4). Control plants were watered to a constant weight with distilled water supplemented with standard Steiner’s medium once a week, immediately after the measurements. The dehydrated plants were not watered for 6 weeks. Due to the large surface area of the leaves, the degree of hydration was monitored at several points on each leaf, using the reflectance coefficient WBI (water band index). In non-destructive analyses (Chl *a* fluorescence, leaf reflectance), a double control was used (measurements at the beginning of the experiment and in the following 6 weeks in watered plants), and the measurements were carried out on 5 plants from each group, on the same leaves. For the measurement of low-temperature fluorescence and proline content, fragments from 3 different leaves were taken in each successive week after dehydration. Measurements were taken immediately or samples were frozen in liquid nitrogen and stored at −70 °C until measurement.

### 4.2. Plant Anatomy

The analysis of the anatomy was performed on microscope slides in distilled water, representing cross-sections of the leaf lamina using a light microscope and a Nikon ECLIPSE Ni epifluorescence microscope (Nikon, Tokyo, Japan) equipped with Microscope Camera Digital Sight series DS-Fi1c and NIS Imaging, Nikon v. 4.11 software. Hand-cut sections were obtained from parts located approximately 2 cm apart from the top of the leaf.

### 4.3. Leaf Reflectance

Leaf reflectance was measured with a CI-710 spectrometer (CID-Science, Camas, WA, USA) on the upper leaf surface at 23 °C. Reflectance spectra in the range of 400–1000 nm were recorded using the SpectraSnap program. Based on the curves, the water band index values were calculated:

WBI = R_900_ × (R_970_)^−1^ (R_x_—is the reflectance intensity at a specific wavelength x) [29].

In addition, the content of selected pigments was estimated based on the following reflectance coefficients: (i) ARI1 = [R_550_^−1^ − R_700_^−1^] × R_800_—anthocyanin pool [44], (ii) CRI1 = [(1/R_510_) − (1/R_550_)]—carotenoid pool [65], (iii) SIPI = [R_800_ − R_445_] × [R_800_ + R_680_]^−1^—ratio of carotenoid concentration to Chl *a* [66], (iv) FRI = [R^−1^_410_ − R^−1^_460_] × R_800_—flavonols pool [67]. The photochemical reflectance index was also determined—PRI = [R_531_ − R_570_] × [R_531_ + R_570_]^−1^ [68].

### 4.4. Chlorophyll a Fluorescence

Chl *a* fluorescence kinetics parameters were measured according to the method of Strasser et al. [49] with use Handy-PEA (Hansatech Instruments, Narborough, UK). Leaf blade fragments were acclimated to darkness for 20–25 min before measurement. Chl *a* FL was induced with 3500 μmol quantum m^−2^ s^−1^ radiation (peak intensity 650 nm, half-width spectral line 22 nm). The following basic parameters of Chl a FL kinetics were analyzed: Fv/Fm—maximum quantum yield of PSII; Fv/F_0_—indicator of structural damage of thylakoids; A_M_—surface area above the OJIP curve and specific energy fluxes expressed per active RC of PSII; ABS/RC—apparent antenna size of active RC; DI_0_/RC—total energy dissipation not trapped by the PSII RC; TR_0_/RC—energy trapping of one active PSII RC; ET0/RC—rate of electron transfer by the active PSII RC; RE_0_/RC—quantum yield of electron transport from Q_A_ to the PSI end electron acceptors. In addition, the performance of electron flux to the final PSI electron acceptors (PI_total_) and quantum yields such as φEo—quantum yield for electron transport from Q_A_− to plastoquinone; φPo—maximum quantum yield of primary PSII photochemistry; and φRo—quantum yield for reduction of end electron acceptors at the PSI acceptor side was also determined.

The OJIP curves were plotted using the following steps: O—20 μs, J—2 ms, I—30 ms, P—300 ms, and then they were normalized to steps O and P. The differential curve for the O–J phase was calculated by subtracting the FL induction curve values obtained for dehydrated plants from control values at each time point (0–6 weeks). The methodology described in [46] was used for the calculations.

### 4.5. Low-Temperature Fluorescence at 77 K

The measurement of the low-temperature fluorescence spectrum (77 K) was performed according to the methodology of Wiciarz et al. [56]. The frozen plant material was homogenized to leaf powder and then mixed with Hepes 0.05 M + 0.33 M sorbitol buffer (pH 7.5) in the proportion of 0.1 g/1 mL. The measurement was performed at 77 K using an LS 55 spectrofluorimeter (Perkin Elmer, Waltham, MA, USA). The sample was placed in a quartz glass capillary and excited with radiation at a wavelength of 437 nm. The fluorescence spectrum was recorded in the range of 650–800 nm using the FL WinLab 4.0 software (Perkin Elmer, Waltham, MA, USA). The curves were normalized to the maximum PSII fluorescence value. Based on the spectra, the values of the F730/F690 ratio were calculated, which allows to estimate the relative share of PSI and PSII in the thylakoid membranes.

### 4.6. Gas Exchange of Leaves

The Pg and R intensity were determined using a Clark electrode (Hansatech Instruments Ltd., Norfolk, UK). The electrode was located in the LD/2 chamber (5 mL volume) at 23 °C and connected to the CB1D data reading device. Data reading and analysis were performed using the Acquire program (Hansatech Group, Norfolk, UK). The PG and R values were determined in a closed system containing 21% of O_2_. The CO_2_ concentration was 300–400 µmol mol^−1^, PFD = 100 µmol m^−2^ s^−1^, temperature 25 °C. The rate of photosynthesis and leaf respiration was expressed in µmol of CO_2_ production/O_2_ taken m^−2^ s^−1^. Stomatal conductance (gs) was measured using the CID CI-340 Handheld Photosynthesis System (CID-Science, Camas, WA, USA) in an open system at a flow rate of 0.5 lpm, in natural light, at 22 °C, using a 6.5 cm^2^ CI-301LC leaf chamber.

### 4.7. Chlorophyll Content

Chl total, Chl *a* and Chl *b* content in the leaves was estimated using the CI-710s spectrometer (CID-Science, Camas, WA, USA). The measurements were made in week 6 after dehydration and in control plants. The Chl *a/b* ratio was calculated on the basis of the obtained data.

### 4.8. Proline Content

The proline content was determined spectrophotometrically in 1 g of homogenized fresh plant material using a 1% ninhydrin solution in 60% acetic acid according to the method of Bates et al. as modified by [69]. The absorbance measurements were made with a spectrophotometer TYP (Thermo Scientific, Waltham, MA, USA).

### 4.9. Statistical Analysis

All results were analyzed in the Statistica 13 program (TIBCO Software, Palo Alto, CA, USA) using a one-way or multifactorial analysis of variance (ANOVA/MANOVA). The significance of the differences between averages was tested using Tukey’s test (HSD) at a significance level of *p* ≤ 0.05.

## 5. Conclusions

Changes in the functioning of the photosynthesis light phase and the pigment composition of leaves are related to the resistance of elkhorn fern to long-term water deficit. *P. bifurcatum*, like other species of epiphytic ferns, has anatomical and physiological adaptations to periodic water shortages, thanks to which even a prolonged period of drought resulted in a slight decrease in leaf hydration with no anatomical and morphological differences. Despite this, disturbances in photosynthesis were observed, which intensified over time. The early physiological response was manifested by abnormalities in the initial phases of the photosynthetic light phase, mainly related to the transport of energy through the LHCII to RC of PSII. This fact should be taken into account in ecophysiological studies on this ecological group of plants, which usually include only gas exchange measurements.

We are currently observing progressing unfavorable changes in water relations in forest ecosystems. For this reason, understanding the mechanisms of water shortage tolerance in as many plant species as possible (including ferns) is very important for the protection of species biodiversity. Future studies on epiphyte photosynthetic apparatus response should, however, include a detailed analysis of the photosynthesis light phase. Data obtained using non-destructive methods allow you to quickly, effectively and at an early stage determine the condition of plants and take actions related to the protection of habitats. In addition, they allow us to learn about the species that are the best tolerant of water deficiency and their possibilities of use in environments changed by human activity.

## Figures and Tables

**Table 1:** Reflectance parameters of *Platycerium bifurcatum* leaves measured following 6 weeks of dehydration (W1–W6) and at the beginning of the experiment (W0). The values for the control plants are shown in Table S1. The parameters’ description: see materials and methods. Mean values from 5 biological replicates marked with different letters in the line differ significantly according to the Tukey’s test, *p* ≤ 0.05.

Parameters	W0	W1	W2	W3	W4	W5	W6
ARI1	0.006 a	0.009 a	−0.004 b	−0.003 b	−0.002 b	0.001 ab	0.002 ab
	±0.002	±0.002	±0.002	±0.001	±0.002	±0.002	±0.002
CRI1	0.161 a	0.155 a	0.072 b	0.084 b	0.095 b	0.088 b	0.111 ab
	±0.025	±0.012	±0.005	±0.007	±0.007	±0.008	±0.008
SIPI	0.868 ab	0.887 a	0.843 b	0.854 b	0.857 ab	0.850 b	0.861 ab
	±0.010	±0.007	±0.005	±0.006	±0.006	±0.006	±0.006
FRI	−1.888 a	−2.947 b	−2.665 ab	−2.535 ab	−2.731 ab	−2.394 ab	−2.845 b
	±0.218	±0.342	±0.135	±0.177	±0.173	±0.187	±0.190
PRI	0.090 a	0.037 d	0.076 ab	0.070 bc	0.077 ab	0.079 ab	0.055 c
	±0.004	±0.005	±0.004	±0.002	±0.004	±0.003	±0.003

**Table 2:** Values of chlorophyll *a* fluorescence parameters of *Platycerium bifurcatum* leaves measured in the following 6 weeks after dehydration (W1–W6) and at the beginning of the experiment (W0). The values for the control plants are shown in Table S2. Mean values from 5 biological replicates marked with different letters in the line differ significantly according to the Tukey’s test, *p* ≤ 0.05.

Parameters	W0	W1	W2	W3	W4	W5	W6
Measured parameters and basic JIP test parameters							
PI total	9.619 a	5.678 b	4.808 bc	4.748 bc	3.483 c	0.771 d	0.321 d
	±0.780	±0.777	±0.488	±0.609	±0.411	±0.056	±0.029
Fv/F_0_	4.802 a	4.656 a	4.003 b	3.940 b	3.811 b	3.912 b	2.454 c
	±0.091	±0.132	±0.121	±0.171	±0.188	±0.099	±0.106
Fv/Fm	0.827 a	0.822 a	0.799 b	0.795 b	0.789 b	0.796 b	0.708 c
	±0.003	±0.004	±0.005	±0.007	±0.008	±0.004	±0.009
A_M_	50,216 a	36,024 bc	33,178 c	43,033 ab	31,311 c	34,245 c	28,701 c
	±3550	±1787	±1886	±3734	±1783	±3380	±2607
Specific Energy fluxes expressed per active RC of PSII							
ABS/RC	1.199 d	1.279 d	1.488 c	1.570 c	1.617 c	1.957 b	2.404 a
	±0.021	±0.046	±0.056	±0.082	±0.084	±0.042	±0.045
DI_0_/RC	0.208 d	0.230 d	0.301 c	0.326 c	0.346 bc	0.401 b	0.706 a
	±0.006	±0.013	±0.018	±0.025	±0.030	±0.015	±0.033
TR_0_/RC	0.992 d	1.050 d	1.186 c	1.244 c	1.271 c	1.556 b	1.698 a
	±0.015	±0.034	±0.040	±0.059	±0.058	±0.029	±0.016
ET_0_/RC	0.735 bc	0.752 b	0.870 a	0.881 a	0.884 a	0.739 bc	0.655 c
	±0.017	±0.031	±0.028	±0.039	±0.036	±0.037	±0.013
RE_0_/RC	0.326 a	0.271 b	0.320 a	0.354 a	0.323 a	0.218 c	0.215 c
	±0.009	±0.014	±0.011	±0.014	±0.018	±0.012	±0.011
Quantum yield parameters							
φPo	0.827 a	0.822 a	0.799 b	0.795 b	0.789 b	0.796 b	0.708 c
	±0.003	±0.004	±0.005	±0.007	±0.008	±0.004	±0.009
φEo	0.613 a	0.588 ab	0.588 ab	0.565 b	0.551 b	0.377 c	0.274 d
	±0.011	±0.012	±0.015	±0.012	±0.013	±0.014	±0.009
φRo	0.272 a	0.212 bc	0.217 bc	0.229 b	0.202 c	0.111 d	0.090 d
	±0.008	±0.008	±0.007	±0.011	±0.011	±0.005	±0.005

## Data Availability

Data are available from the authors upon request.

## Supplementary Materials

The following supporting information can be downloaded at https://www.mdpi.com/article/10.3390/ijms241512064/s1.
