# Supplementary material for: Water-Deficit Stress in the Epiphytic Elkhorn Fern: Insight into Photosynthetic Response
Source: Int J Mol Sci. 2023 Jul 27;24(15):12064. doi: 10.3390/ijms241512064 (PMC10418323; doi:10.3390/ijms241512064)
Supplement: Supplementary file 1 [file ijms-24-12064-s001.zip › ijms-2501138-supplementary.pdf]

Supplementary materials

Figure S1. Reflectance curves in *Platycerium bifurcatum* leaves: A - average values at the beginning of the experiment and 6 week after dehydration, B - average values in control plants at the beginning and end of the experiment. Mean values of 5 biological replicates for each treatment

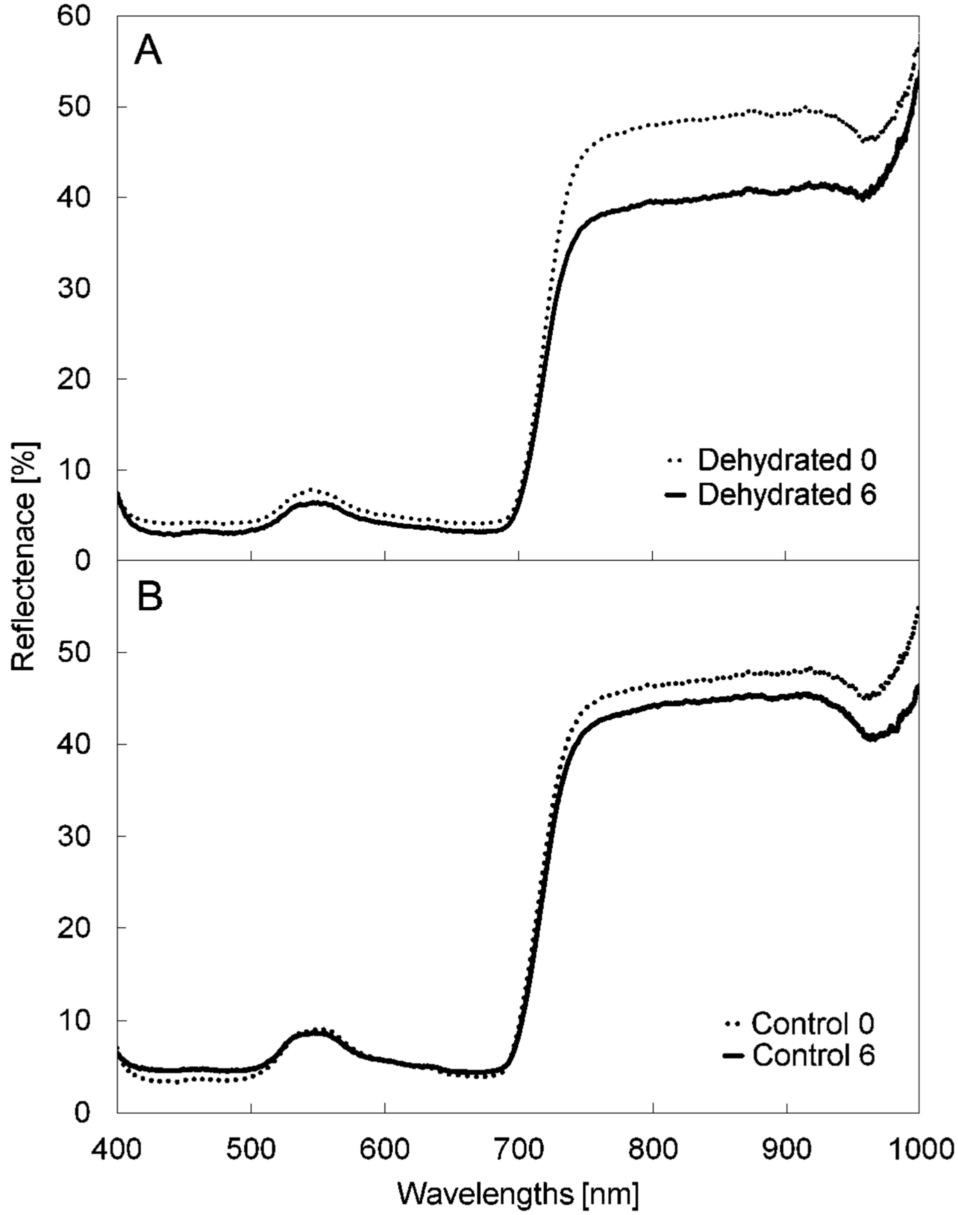

Figure S2. Chlorophyll *a* fluorescence induction curves (OJIP) in *Platycerium bifurcatum* leaves: A - in the following 6 weeks after dehydration and B - in control plants (at the beginning and in the following 6 weeks of the experiment. Mean values of 5 biological replicates for each treatment

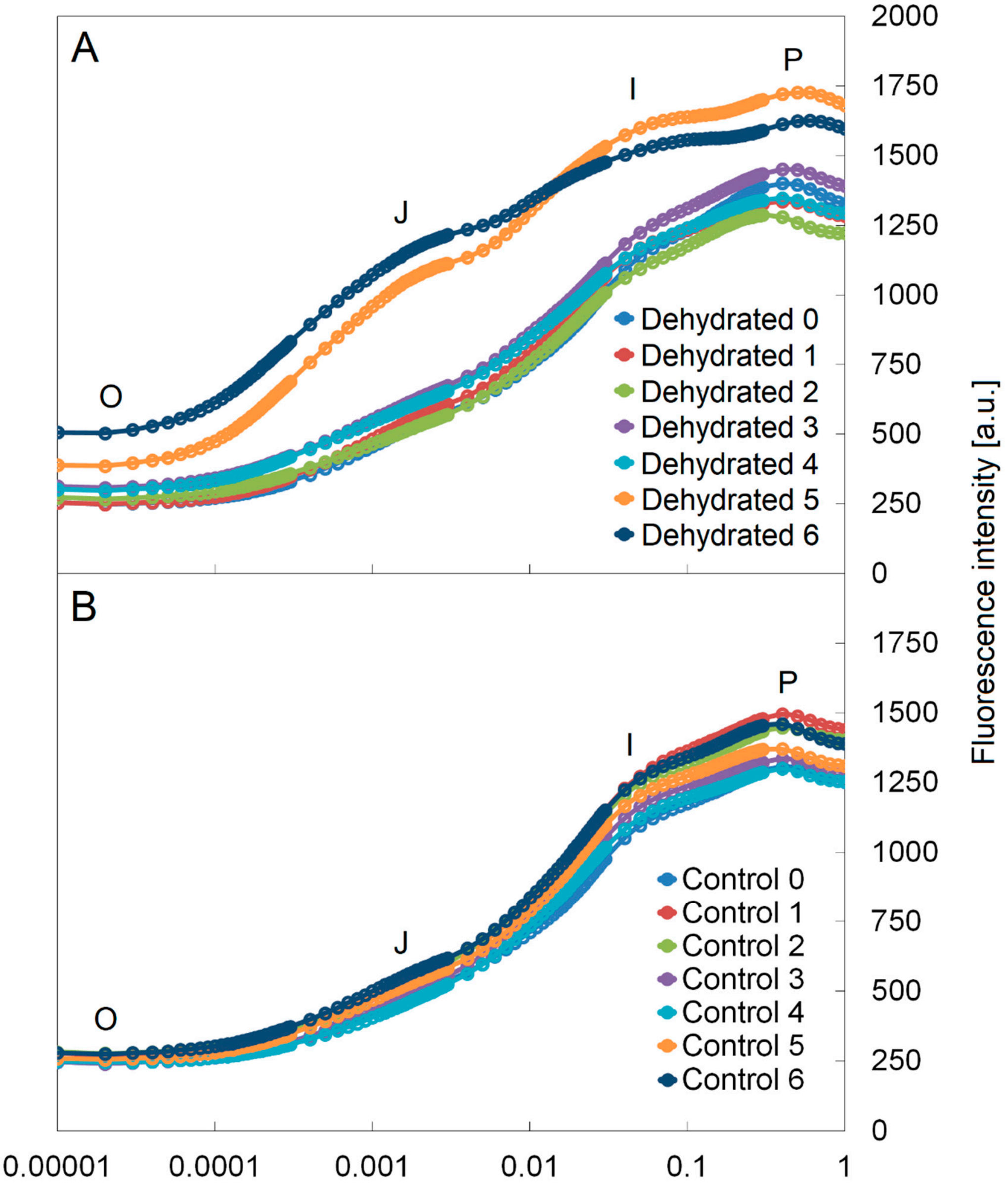

Figure S3. Low-temperature fluorescence curves at 77K in *Platycerium bifurcatum* leaves:
A - in the following 6 weeks after dehydration and B - in control plants (at the beginning and in the following 6 weeks of the experiment. Mean values of 5 biological replicates for each treatment

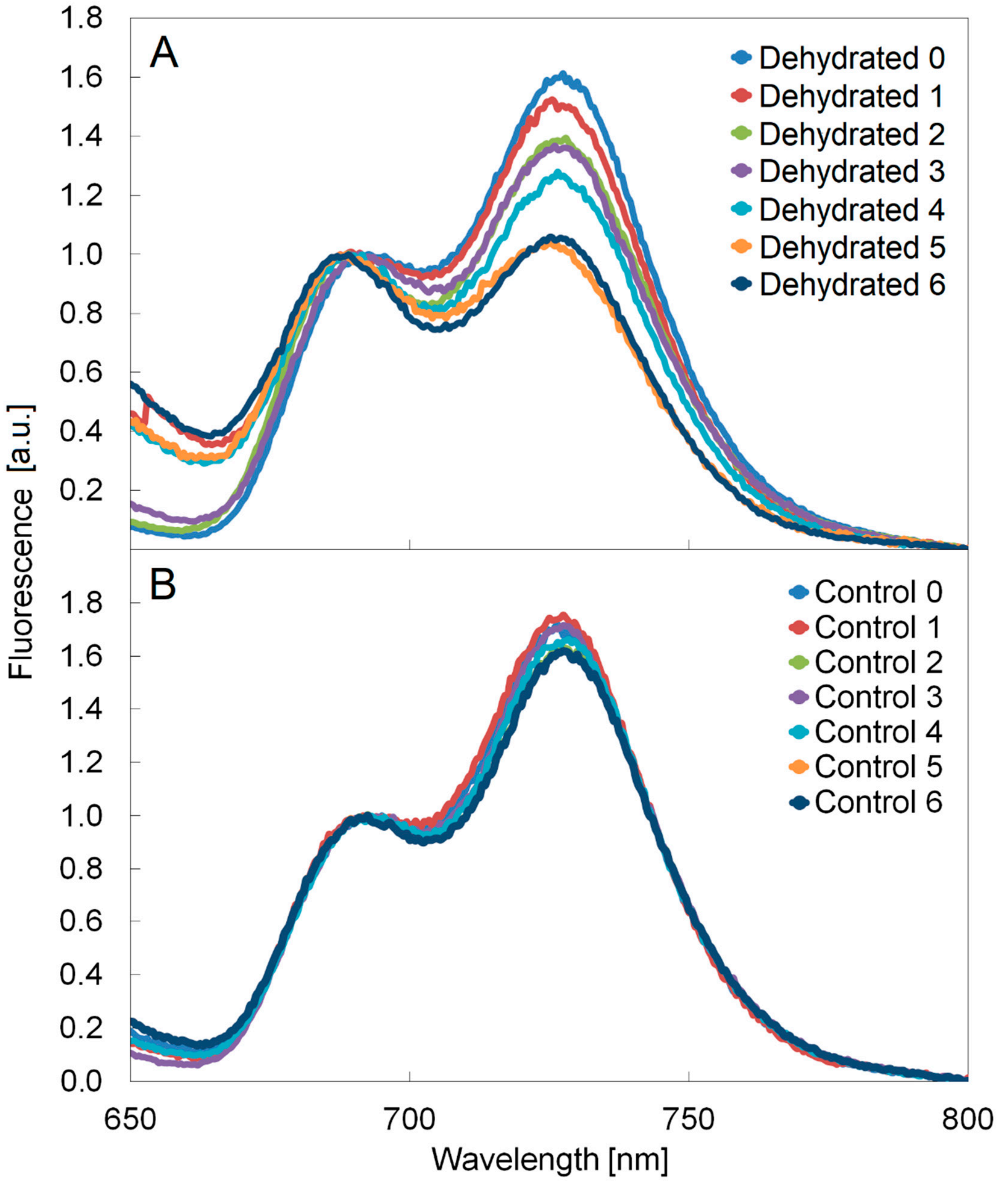

Table S1. Reflectance parameters of *Platycerium bifurcatum* leaves under control conditions for 6 weeks (W1-W6) and at the beginning of the experiment (W0). Mean values from 5 biological replicates marked with different letters in the line differ significantly according to the Tukey's test, $p \leq 0.05$

**Parameters**	**W0**	**W1**	**W2**	**W3**	**W4**	**W5**	**W6**
ARI1	0.006a	0.002a	0.001a	0.003a	0.004a	0.005a	0.006a
	±0.002	±0.002	±0.003	±0.002	±0.002	±0.003	±0.002
CRI1	0.162a	0.156a	0.108a	0.109a	0.115a	0.110a	0.107a
	±0.025	±0.023	±0.008	±0.009	±0.008	±0.009	±0.008
SIPI	0.868a	0.878a	0.857a	0.853a	0.856a	0.850a	0.858a
	±0.010	±0.009	±0.007	±0.006	±0.007	±0.007	±0.006
FRI	-1.883a	-2.178a	-1.766a	-1.916a	-2.151a	-1.875a	-2.094a
	±0.216	±0.458	±0.247	±0.216	±0.195	±0.205	±0.206
PRI	0.088a	0.063bc	0.080ab	0.069ab	0.069ab	0.068ab	0.042c
	±0.004	±0.006	±0.004	±0.006	±0.004	±0.007	±0.005

Table S2. Values of chlorophyll *a* fluorescence parameters of *Platycerium bifurcatum* leaves under control conditions for 6 weeks (W1-W6) and at the beginning of the experiment (W0). Mean values from 5 biological replicates marked with different letters in the line differ significantly according to the Tukey's test, $p \leq 0.05$

**Parameters**	**W0**	**W1**	**W2**	**W3**	**W4**	**W5**	**W6**
Measured parameters and basic JIP-test parameters							
PI_{total}	8.450a	7.364ab	5.877b	6.859ab	6.546ab	4.988b	5.083b
	±1.284	±0.729	±0.966	±0.962	±0.852	±0.308	±0.362
Fv/F_0	4,556a	4,639a	4,381a	4,613a	4,384a	4,608a	4,523a
	±0,123	±0,195	±0,216	±0,262	±0,341	±0,110	±0,125
Fv/Fm	0.819a	0.826a	0.811a	0.824a	0.806a	0.821a	0.818a
	±0.004	±0.004	±0.008	±0.006	±0.014	±0.004	±0.004
A_M	47228a	43852ab	41567ab	36197b	34272b	38784ab	39412ab
	±3908	±3758	±4355	±2587	±2899	±2937	±3289
Specific Energy fluxes expressed per active RC of PSII							
ABS/RC	1.295a	1.235a	1.360a	1.237a	1.250a	1.400a	1.412a
	±0.039	±0.043	±0.066	±0.063	±0.068	±0.049	±0.056
DI_0/RC	0.235a	0.224a	0.262a	0.233a	0.250a	0.252a	0.259a
	±0.011	±0.015	±0.023	±0.024	±0.032	±0.013	±0.015
TR_0/RC	1.060ab	1.011b	1.099ab	1.004b	1.000b	1.147a	1.153a
	±0.029	±0.030	±0.044	±0.040	±0.039	±0.037	±0.041
ET_0/RC	0.801ab	0.774ab	0.830ab	0.755b	0.767ab	0.842ab	0.854a
	±0.016	±0.024	±0.029	±0.031	±0.032	±0.031	±0.038
RE_0/RC	0.324a	0.282a	0.283a	0.266a	0.269a	0.295a	0.303a
	±0.015	±0.014	±0.021	±0.024	±0.023	±0.018	±0.021
Quantum yields parameters							
φPo	0.819a	0.821a	0.811a	0.817a	0.806a	0.821a	0.818a
	±0.004	±0.006	±0.008	±0.010	±0.014	±0.004	±0.004
φEo	0.621a	0.629a	0.615a	0.615a	0.618a	0.602a	0.604a
	±0.011	±0.010	±0.014	±0.013	±0.016	±0.009	±0.010
φRo	0.252a	0.228ab	0.208b	0.214b	0.213b	0.209b	0.212b
	±0.015	±0.008	±0.014	±0.014	±0.012	±0.007	±0.009
